# Recommended X-ray single-crystal structure refinement and Rietveld refinement procedure for tremolite

**DOI:** 10.1107/S2052520621004844

**Published:** 2021-07-14

**Authors:** Paolo Ballirano, Beatrice Celata, Alessandro Pacella, Ferdinando Bosi

**Affiliations:** a Dipartimento di Scienze della Terra, Sapienza Università di Roma, Piazzale Aldo Moro 5, Rome, I-00185, Italy

**Keywords:** structure refinement, Rietveld method, scattering curves, tremolite, asbestos

## Abstract

A detailed description of the structure of the amphibole-supergroup minerals is very challenging owing to their complex chemical composition that renders the process of cation partition extremely difficult, particularly because of the occurrence of multivalent elements. Since amphiboles naturally occur under a fibrous morphology and have largely been used to produce asbestos, there is a growing demand for detailed and accurate structural data in order to study the relationships between structure, composition and toxicity. The present study proposes a recommended refinement procedure for both X-ray single-crystal structure refinement (SREF) and Rietveld analysis for tremolite, selected as a test case. The corresponding structural results are compared to estimate the ‘degree of confidence’ of the Rietveld refinement with regard to SREF. In particular, it is shown that the interpretation of the electron density of the tremolite structure by SREF is model dependent. By assuming that the site-scattering values from SREF should be as close as possible to those from electron microprobe analysis, as a crucial constraint for the correct description of the final crystal-chemical model, it is found that it is best satisfied by using partially ionized scattering curves (SCs) for O and Si, and neutral SCs (neutral oxygen curves or NOCs) for other atoms. This combination leads to the best fit to the diffraction data. Moreover, it is found that Rietveld refinement using NOCs produces the best structural results, in excellent agreement with SREF. It is worth noting that, due to the complexity of the diffraction pattern and the fairly large number of freely refinable parameters, refinements with different combinations of SCs produce results almost indistinguishable from a statistical point of view, albeit showing significant differences from a structural point of view.

## Abstract 2

This study aims to solve the site-occupancy issues of any crystalline substance, showing that the structure refinement results should not be limited to a simple description of the crystal geometry but should go beyond that, providing refined site-scattering values consistent with the chemical data.

## 1. Introduction   

Amphiboles are among the most thoroughly investigated minerals from a structural and crystal-chemical viewpoint. Large crystal-structure databases have been built and extended modelling of the mutual crystal-chemical and structural relations have been developed (*e.g.* Hawthorne & Oberti, 2007 ▸). The detailed modelling depends not only on the bond distances, but also on the ability to quantify as accurately as possible the site scattering (SS) power at the structural sites, because it is an important piece of information in the assignment of complex site populations involving various different atoms.

However, the observed SS values depend heavily on the strategy used during structure refinements (SREFs) on X-ray single-crystal diffraction (XRSCD) data. In particular, results can be affected by the use of different types of X-ray scattering curves (SCs): neutral, or partially or fully ionized for anions and cations. It is worth noting that the choice of (partially) ionized SCs may significantly affect the number of electrons in the crystal unit cell at θ = 0°, *F*(000), and the scale factor in least-squares refinement. This is due to the fact that their use implies a different number of electrons at θ = 0° affecting the correlation between displacement parameters and scale factor (Coppens, 1997 ▸). Nevertheless, the use of different combinations of SCs has been commonly used to compensate empirically for perturbation of the electron density caused by interaction with other atoms in cases where more refined approaches are not applicable (Coppens, 1997 ▸). This is promoted by the fact that the scattering of the atomic valence electrons is concentrated in the low-order region of reciprocal space, *i.e.* in the diffraction zone sinθ/λ < 0.25 Å^−1^, where neutral and ionized SCs differ more significantly.

In order to provide consistent results, the type of SC used in the refinement should be clearly stated by the researchers, but such information is instead often missing from the related papers. Hawthorne *et al.* (1995 ▸) have briefly summarized the results of extensive methodological tests performed at the Centro di Studio per la Cristallochimica e la Cristallografia, Pavia, Italy, on *ca* 1500 rock-forming minerals, indicating that the most accurate results in terms of SS are obtained by ‘*…(1) using ionized X-ray scattering curves for non-tetrahedral cations and (2) refining the occupancy of ionized versus neutral species for anions (O^2−^ versus O) and tetrahedral cations (Si_*x*_^4+^Al_1–*x*_^3+^ versus Si_*x*_Al_1–*x*_)…*’. These authors also observed that the refined values for the Si formal charge between +1 and +2 are a nice indication for the strong co­valent character of the Si—O bond.

Nevertheless, to the best of our knowledge, an in-depth systematic and quantitative analysis of the effect of the scattering factor types used for the atoms on the refined SS values, as well as on other refined structural parameters (*e.g.* displace­ment parameters and bond distances), is missing for amphiboles.

In recent years, fibrous amphiboles have attracted increased interest due to their environmental and health relevance (Ballirano *et al.*, 2017 ▸). In fact, fibrous amphiboles are naturally occurring asbestos and common constituents or contaminants of asbestos-containing materials as well. The commercial term ‘asbestos’ groups together five fibrous amphiboles [actinolite, ‘amosite’ (fibrous variety of grunerite), anthophyllite, ‘crocidolite’ (fibrous variety of riebeckite) and tremolite] and one fibrous serpentine mineral, chrysotile (IARC, 2012 ▸). In addition, recent epidemiological studies have revealed some cases of environmental contamination from unregulated fibrous amphiboles in both Italy and the USA (Paoletti *et al.*, 2000 ▸; Wylie & Verkouteren, 2000 ▸; Erskine & Bailey, 2018 ▸; Laurita & Rizzo, 2019 ▸).

In order to unravel possible relations between structure, composition and toxicity, detailed and accurate structural data are needed. However, the structural analysis of fibrous amphiboles poses additional complications compared with that of prismatic samples having adequate dimensions for SREF. In fact, despite the promising attempts to use synchrotron nano-diffraction experiments (Giacobbe *et al.*, 2018 ▸), Rietveld refinements on X-ray powder diffraction (XRPD) data still represent the best method for retrieving structural information from fibrous amphiboles. In detail, the transmission mode using a capillary is the experimental setup for XRPD data collection that is particularly well suited to this task, being very successful at reducing the parasitic effects of preferred orientation. Several studies have been addressed to such an experimental setup for analysing the structure of fibrous amphiboles, not only under ambient conditions (Gianfagna *et al.*, 2007 ▸; Andreozzi *et al.*, 2009 ▸; Vignaroli *et al.*, 2014 ▸; Pacella *et al.*, 2019 ▸; Ballirano & Pacella, 2020 ▸) but also investigating *in situ* the structural modifications induced by high temperatures (Pacella *et al.*, 2020 ▸; Ballirano & Pacella, 2020 ▸). In particular, the refined structural data [bond distances (BD) and SS] were fully consistent with chemical composition, providing meaningful correlations with different ongoing processes (cation exchange, Fe oxidation *etc.*) whose onsets were confirmed by means of other analytical tools. However, to date and to the best of our knowledge, there is no analysis of the ‘confidence level’ of such results compared with those arising from single-crystal SREF. Despite the large wealth of papers addressing this general topic (XRPD versus XRSCD data refinement), none of them has been especially devoted to amphiboles.

This study aims to show that the most accurate results for the determination of site populations are only obtained where the most appropriate scattering curves (derived from neutral versus ionized atoms) are used to obtain total SS values that best match the chemical data. In order to provide relevant information to perform an optimal SREF, the present study is addressed to a systematic analysis of the effect of different types of scattering curves used for the atoms on the refined structural parameters of a gem-quality tremolite sample from Merelani (Tanzania). This sample was selected as a case study for its high crystallinity and chemical homogeneity. The SREF results were then compared with those obtained from Rietveld refinement in order to estimate their ‘degree of confidence’. Similarly, in the case of Rietveld refinements, the effects of choosing different SCs have been investigated to identify the best computational conditions, as already done for the spinel structure by Ballirano (2003 ▸). The results of the present study should be intended as representing the most appropriate empirical procedure for modelling the electron-density distribution in minerals having no claim for rigorous justification(s) based on the nature of the chemical bond.

## 2. Amphibole formulae   

Minerals are chemical substances that need to be represented by formulae. There are several types of mineral formulae (*e.g.* Bosi *et al.*, 2019 ▸), but for the purposes of this study we are interested in the structural and chemical ones.

The general *structural* formula of an amphibole may be written as *AM*(4)_2_[*M*(1)_2_ *M*(2)_2_ *M*(3)][*T*(1)_4_ *T*(2)_4_]O_22_O(3)_2_, where the italicized letters represent non-equivalent structural sites.

The general *chemical* formula of the amphibole-supergroup minerals may be written as AB_2_C_5_T_8_O_22_W_2_, where:

A = □, Na, K, Ca, Pb, Li;

B = Na, Ca, Mn^2+^, Fe^2+^, Mg, Li;

C = Mg, Fe^2+^, Mn^2+^, Al, Fe^3+^, Mn^3+^, Cr^3+^, Ti^4+^, Li;

T = Si, Al, Ti^4+^, Be;

W = (OH), F, Cl, O^2–^.

Note that the non-italicized letters represent groups of ions, such as the C cations that occur at the octahedrally coordinated *M*(1,2,3) sites or the W anions at the O(3) site (Hawthorne & Oberti, 2007 ▸).

## 3. Experimental   

### 3.1. Microchemical data   

A yellowish–green amphibole crystal fragment, previously used for SREF (see below), was chemically characterized by electron microprobe analysis (EMPA). This analysis was obtained using a wavelength-dispersive spectrometer (WDS mode) with a Cameca SX50 instrument at the CNR Istituto di Geologia Ambientale e Geoingegneria (Rome, Italy), operating at an accelerating potential of 15 kV with a 15 nA current and a 1 µm beam diameter. Minerals and synthetic compounds were used as standards: wollastonite (Si, Ca), magnetite (Fe), rutile (Ti), corundum (Al), vanadinite (V), fluorphlogopite (F), periclase (Mg), jadeite (Na), orthoclase (K), sphalerite (Zn), rhodonite (Mn) and metallic Cr. The *PAP* correction procedure for quantitative electron probe microanalysis was applied (Pouchou & Pichoir, 1991 ▸). The results are reported in Table 1 ▸ and represent the mean values of 15 spot analyses across the crystal used for SREF study. Zinc was below its respective detection limit (0.03 wt%) in the studied sample.

### 3.2. XRSCD and SREF   

A representative crystal fragment (0.21 × 0.22 × 0.31 mm) of the Merelani amphibole was selected for X-ray diffraction measurements on a Bruker KAPPA APEXII single-crystal diffractometer (Earth Sciences Department, Sapienza University of Rome), equipped with a CCD area detector (6.2 × 6.2 cm active detection area, 512 × 512 pixels) and a graphite crystal monochromator, using Mo*K*α radiation from a fine-focus sealed X-ray tube. The sample-to-detector distance was 4 cm. A total of 3687 exposures (step = 0.2°, time per step = 20 s) covering a full reciprocal sphere with a completeness of 99.9% and redundancy of approximately 7 were collected. Final unit-cell parameters were refined using the *SAINT* program (Bruker, 2016 ▸) on 9978 reflections with *I* > 10σ(*I*) in the range 8° < 2θ < 81°: *a* = 9.8348 (3) Å, *b* = 18.0035 (6) Å, *c* = 5.2825 (2) Å, β = 104.9961 (8)° and *V* = 903.47 (2) Å^3^. The associated intensities were processed and corrected for Lorentz and background effects plus polarization, using the *APEX2* software program (Bruker, 2016 ▸). The data were corrected for absorption using a multi-scan method (*SADABS*; Bruker, 2016 ▸). The absorption correction led to a significant improvement in *wR*_2, int_ (from 0.0358 to 0.0219). No violation of *C*2/*m* symmetry was detected.

Structure refinement was done using the *SHELXL2013* program (Sheldrick, 2015 ▸) coupled with the Qt64 graphical user interface *SHELXLE* (Hübschle *et al.*, 2011 ▸). Starting coordinates were taken from Evans & Yang (1998 ▸). Variable parameters were: scale factor, extinction coefficient, atom coordinates, site-scattering values and atomic displacement factors. Regarding the atomic model refinement, the *A* site, actually *A*(*m*), was modelled using the K/K^+^ scattering factor. The occupancies of the *M*(1,2,3) and *M*(4) sites were obtained considering the presence of Mg/Mg^2+^ and Ca/Ca^2+^ scattering factors, respectively. The *T*(1) site was first modelled with Si/Si^4+^ scattering factors, and subsequently its occupancy was fixed to [(Si versus Si^4+^)_0.83_Al_0.17_)] = 1.00, that is, the value obtained from the empirical structural formula (see below) and with the Al scattering factor. The *T*(2) site was modelled with Si/Si^4+^ scattering factors and a fixed occupancy of 1. The anion sites were modelled with O/O^1−^/O^2−^ scattering factors and a fixed occupancy of 1. A final refinement was then done by modelling the site occupancy of the O(3) site with O and F fixed to the values obtained from the empirical formula (see below). Hydrogen site occupancy was also constrained to be equal to the O one.

A key issue in such an atomic model is the type of scattering factors used for the refinement. In detail, nine different SREFs were done using the following combinations:

(*a*) Neutral O (O^0^) SC (hereinafter termed NOC, Neutral Oxygen scattering Curve);

(*b*) O^0^ and fully ionized SCs of cations at the *M* (Mg^2+^, Ca^2+^) and *A* sites (K^+^);

(*c*) O^1−^ SC;

(*d*) O^2−^ SC;

(*e*) Optimized O^*x*−^ SC arising from:

(i) O^2−^ versus O^1−^ SC refinement and

(ii) O^2−^ versus O^0^ SC refinement (hereinafter termed OOC, Optimized Oxygen scattering Curve);

(*f*) Optimized O^*x*−^ and Si^*x*+^ SCs in the case of

(i) O^2−^ versus O^1−^ SC refinement and

(ii) O^2−^ versus O^0^ SC refinement (hereinafter termed OOSC, Optimized Oxygen and Silicon scattering Curve).

(*g*) Optimized O^*x*−^, Si^*x*+^ SCs and fully ionized SCs of cations at the *M* (Mg^2+^, Ca^2+^) and *A* sites (K^1+^) in the case of

(i) O^2−^ versus O^1−^ SC refinement and

(ii) O^2−^ versus O^0^ SC refinement.

Optimization was achieved by refining the occupancy of ionized versus ionized or neutral scattering curves for oxygen (O^2−^ versus O^1−^ or O^0^), and ionized versus neutral species for cations at *T* (Si^4+^ versus Si^0^) through the FVAR instruction in *SHELXL2013*. The coefficients for analytical approximation to the scattering factors were taken from Table 6.1.1.4 of the *International Tables for Crystallography* (Brown *et al.*, 2006 ▸), except for O^2−^, whose coefficients were from Hovestreydt (1983 ▸).

The starting structural model included a single *A*(*m*) site, whose anisotropic displacement parameters were refined to reasonable values. Moreover, in the electron-density synthesis with coefficients (*F*_o_ − *F*_c_), all SREFs showed highest peaks (0.6–0.7 e^−^ Å^−3^) at *ca* (0, 0.462, 0), corresponding to the position of the *A*(2) site in the amphibole structure. However, refinements with the *A* site split into *A*(*m*) and *A*(2) failed. Therefore, the *A* sites were modelled only as an *A*(*m*) site. Finally, a smaller peak (0.5 e^−^ Å^−3^) was also observed at (0, 0.246, 1/2), corresponding to a split *M*(4)′ site, but any attempt to refine it failed. It should be noted that the presence of *M*(4)′ is strictly related to the ^B^(Mg, Fe^2+^, Mn^2+^) constituents (Oberti & Ghose, 1993 ▸; Oberti *et al.*, 1993 ▸, 2006 ▸) which in the studied sample corresponds to ^B^(Mn + Fe^2+^) = 0.023 a.p.f.u. (atoms per formula unit), as reported in the present chemical formula.

Table 2 reports statistical indicators, the highest peak and deepest hole in the difference Fourier synthesis, ionic charges (ICs) for O and Si, and SS at the *M*(1,2,3,4) and *A* sites obtained as a function of the different combinations of SCs. Table 3 lists the variation in equivalent and isotropic displace­ment parameters for the various SREFs. Relevant bond distances are reported in Table 4 and the results of the cation site population in Table 5. Finally for the single-crystal data, Table 6 lists statistical indicators, the highest peak and deepest hole from the electron-density synthesis with coefficients (*F*_o_ − *F*_c_), ionic charges (ICs) for O and Si, and site scattering (SS) of tremolite as a function of different 2θ_max_ from OOSC SREF.

### 3.3. X-ray powder diffraction and Rietveld refinement   

X-ray powder diffraction (XRPD) data were collected on a Bruker AXS D8 Advance instrument operating in θ/θ geometry in transmission mode. The instrument is equipped with focusing multilayer graded (Göbel) mirrors located along the incident beam and Soller slits on both incident (2.3° opening angle) and diffracted (radial) beams. Intensities were collected using a VÅntec-1 position-sensitive detector (PSD) set at an opening angle of 6° 2θ. A fragment of the large crystal of tremolite was selected for XRPD. The fragment was gently ground, in ethanol, in an agate mortar and the resulting powder was loaded into two 0.7 mm diameter borosilicate-glass capillaries that were fixed to a standard goniometer head and aligned. Two capillaries were prepared to check for data collection reproducibility. Data were collected in the 5–145° 2θ angular range, with a 0.0218° 2θ step size and 10 s counting time using Cu *K*α[Cu *K*α1] radiation.

**Figure 1:**
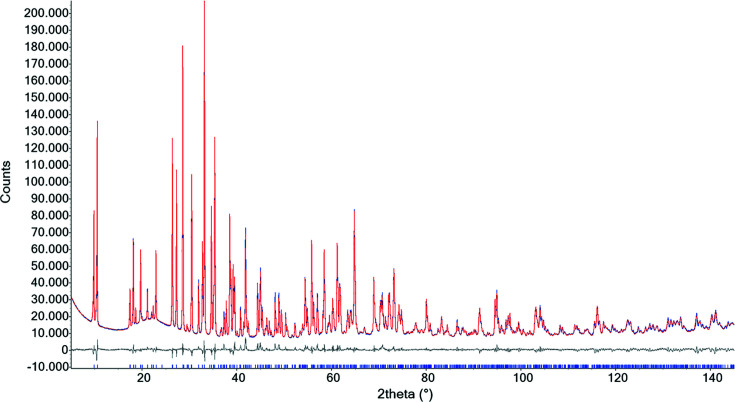
A representative example of a Rietveld plot for the refinement of tremolite using neutral scattering curves. Blue denotes experimental data, red the calculated profile, grey the difference plot and blue vertical bars indicate the position of the calculated Bragg reflections.

Rietveld structure refinement was performed using *TOPAS6* (Bruker, 2016 ▸) which implements the fundamental parameters approach (*FPA*; Cheary & Coelho, 1992 ▸) to describe the peak shape. The occurrence of anisotropic line broadening was detected and modelled using the multi-dimensional distribution of lattice metrics developed by Stephens (1999 ▸). The equation of Sabine *et al.* (1998 ▸) for a cylindrical sample was used for absorption correction. Preferred orientation effects were corrected using normalized symmetrized spherical harmonics functions, as described by Järvinen (1993 ▸), by selecting the number of appropriate terms (sixth order, 15 refinable parameters) as indicated by Ballirano (2003 ▸). Starting structural data were those from the NOC SREF. Isotropic displacement parameters were kept fixed to the values obtained from SREF. Several refinements were performed comprising, among the others, the same combinations explored for SREF. In particular, OOC and OOSC were fixed to the same ICs obtained from SREF for both oxygen and silicon. Average values of the spherical harmonics coefficients used for preferred orientation correction are reported in Table 7. Relevant parameters of the various refinements are listed in Table 8 and a representative example of a Rietveld plot for the NOC refinement is shown in Fig. 1 ▸.

## 4. Results and discussion   

### 4.1. EMPA   

The present yellowish–green amphibole sample from Merelani (Tanzania) is chemically homogeneous, as indicated by the small standard deviation values of the EMPA data (Table 1 ▸). Crystal-chemical considerations indicate that the oxidation state of all Mn and Fe atoms can be assumed to be +2. Moreover, their very low concentrations (<0.20 wt%) are consistent with their occurrence at the *M*(4)′ site, in agreement with SREF. On the basis of the SREF information (see below), Ti^4+^ can be identified as a C cation and allocated at the *M*(1) site. This assignment requires the application of the substitution mechanism ^*M*(1)^Ti^4+^ + 2^W^O^2−^ → ^*M*(1)^(Mg, Fe^2+^) + 2^W^(OH)^−^ that reduces the amount of (OH) at the O(3) site (Hawthorne *et al.*, 2012 ▸). Consequently, due to the presence of Ti^4+^, the H_2_O content and the atomic fractions were calculated by charge balance under the assumption of ^W^(O + OH + F) = 2.000 a.p.f.u. and 24 anions (Table 1 ▸).

In accordance with Hawthorne *et al.* (2012 ▸), the resulting empirical chemical formula is:

^A^(Na_0.185_K_0.105_)_Σ0.290_^B^(Ca_1.945_Mn^2+^_0.016_Mg_0.032_Fe^2+^_0.007_)_Σ2.000_^C^(Mg_4.580_Al_0.343_V_0.040_Cr_0.007_Ti_0.030_)_Σ5.000_^T^(Si_7.320_Al_0.680_)_Σ8.000_ O_22_^W^(OH_1.609_F_0.331_O_0.060_)_Σ2.000_.

This composition corresponds to tremolite, ideally Ca_2_Mg_5_Si_8_O_22_(OH)_2_, with *ca* 30% component of fluorpargasite, ideally NaCa_2_(Mg_4_Al)(Si_6_Al_2_)O_22_(F)_2_.

Based on the SREF information and optimized cation distributions over the *M*(1,2,3) sites (Table 5), the following empirical structural formula is proposed:

*^A^* (Na_0.185_K_0.105_)_Σ0.290_^*M*(4)^(Ca_1.945_Mn^2+^_0.016_Mg_0.032_Fe^2+^_0.007_)_Σ2.000_ [^*M*(1)^(Mg_1.940_Al_0.030_Ti_0.030_)_Σ2.000_^*M*(2)^(Mg_1.740_Al_0.213_V_0.040_Cr_0.007_)_Σ2.000_^*M*(3)^(Mg_0.900_Al_0.100_)_Σ1.000_] [^*T*(1)^(Si_3.320_Al_0.680_)_Σ4.000_^*T*(2)^(Si_4.000_)] O_22_^O(3)^(OH_1.609_F_0.331_O_0.060_)_Σ2.000_.

It is worth noting that the total A-site occupancy approximately equals the F content. This is related to short-range-order constraints involving O(3) and *A* sites (Hawthorne & Della Ventura, 2007 ▸).

A very similar gem-like tremolite sample, consisting of a large centimetric crystal from Merelani (Tanzania), was also characterized by Fritz *et al.* (2007 ▸). These authors reported a very similar chemical composition and suggested, on the basis of optical absorption spectroscopy data, that its yellowish–green colour is caused by V^3+^ and Cr^3+^.

### 4.2. Single-crystal SREFs   

**Figure 2:**
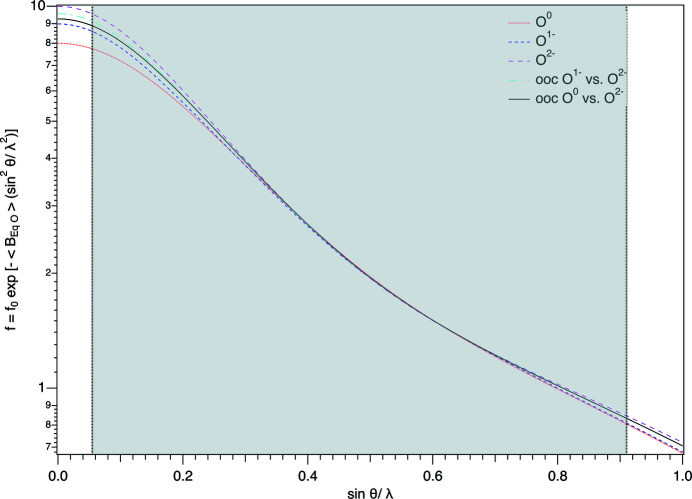
The sin θ/λ dependence of the various scattering curves of oxygen (O^0^, O^1−^, O^2−^, OOC), corrected for the mean displacement parameter of the oxygen sites, used in the various SREFs of tremolite. Data are plotted from the coefficients for analytical approximation to the scattering factors listed for O^0^ and O^1−^ in Table 6.1.1.4 of the *International Tables for Crystallography* (Brown *et al.*, 2006 ▸), and for O^2−^ as given by Hovestreydt (1983 ▸). OOC denotes optimization of O^1−^ versus O^2−^ and O^0^ versus O^2−^ as obtained from SREF (see text for explanation).

Fig. 2 ▸ shows the dependence of the different types of oxygen SCs on sinθ/λ, corrected for their average *U*_eq_ (Debye–Waller factor), that were used in the present SREF. The shaded area delineates the extension of the experimental data set collected, which spans from *ca* 0.055 to 0.91 Å^−1^. As can be seen, differences between the various curves occur at both ends of the range.

Attempts to refine the *T*(1) site population for models (*a*)–(*e*) led to significantly different results, as shown by the Si amounts varying from 2.3 (1) a.p.f.u. for the NOC model to 3.48 (9) a.p.f.u. for the O^2−^ model and to 3.08 (8) a.p.f.u. for the OOC model. Owing to the occurrence of correlations larger than 0.8 between the refined site occupancy factors and displacement parameters in the correlation matrix of *SHELXL*, the population at *T*(1) was fixed to that of the structural formula. As far as the population at *T*(2) is concerned, we decided to consider this site as occupied by Si as no conclusive evidence was observed pointing towards the presence of ^*T*(2)^Al.

From a statistical point of view, the various refinements were characterized by *wR*_2_ values ranging from 0.0314 to 0.0517. The highest *wR*_2_ indices (>0.0373) are associated with models (*a*), (*b*), (*c*) and (*d*), whereas models (*e*), (*f*) and (*g*) have smaller *wR*_2_ values (<0.0347), despite the different combinations of SCs used (Table 2 ▸).

The two largest correlation matrix elements observed consistently in all the SREFs (0.71 and 0.79) only regarded the displacement parameters *U* ^13^ and *U* ^11^/*U* ^33^ of the *A*(*m*) site. As a consequence, no significant correlation occurred between the O and Si SC optimization parameters of FVAR, nor between other structural parameters such as site occupancy factors (SOFs). As a further proof, all the SREFs were repeated after significant modification of the starting parameters, obtaining convergence toward a unequivocal minimum.

Analysis of the *most disagreeable reflection* listed in *SHELXL* (Table 2 ▸) indicated that only a simultaneous optimization of O and Si SCs produces a drastic reduction of both the maximum and the averaged values of the error/s.u. (average calculated over the ten highest disagreeable reflections). Coherently, the average resolution of the most disagreeable reflections tends to reduce, indicating a progressive improvement in the fit of the reflections having small sinθ/λ values, in correspondence of which the scattering contribution of the atomic valence electrons is concentrated.

The use of fully ionized SCs of cations at the *M* (Mg^2+^, Ca^2+^) and *A* (K^+^) sites, as in model (*b*) associated with neutral O and Si SCs and model (*g*) coupled with optimized O and Si SCs, resulted in SS values almost identical to those obtained with neutral SCs, except for the *A*(*m*) site. In that case, fully ionized SCs produced a small increase in the SS of *ca* 0.1 e^−^ (Table 2 ▸).

The NOC SREF structural results were characterized by SS at the *M* and *A* sites slightly lower than those obtained by the chemical data: *ca* 1.2, 0.6 and 0.3 e^−^ at *M*(1,2,3), *M*(4) and *A*(*m*), respectively. Switching from O^0^ to O^1−^ and to O^2−^ SCs [models (*c*) and (*d*), respectively] led to a progressive decrease in the *wR*_2_ values along with a regular increase, by *ca* 3% and 7%, in the SS at the *M*(1,2,3,4) and *A*(*m*) sites, respectively. However, in model (*d*) the SS values at *M*(1,2,3) were 0.9–1.3% higher than those obtained from the chemical formula (Table 2 ▸).

A significantly better fit with the chemical data was obtained for model (*e*), but a further improvement in terms of *R* indices was reached for model (*f*), in particular for O^2−^ versus O^0^ SC optimization (OOSC), yielding the lowest *wR*_2_ value of 0.0314.

The latter converged to ICs for O and Si of −1.288 and +0.887, respectively. As a general observation, refinement of O^2−^ versus O^0^ instead of O^2−^ versus O^1−^ in both models (*e*) and (*f*) not only produced a marginal reduction in *wR*_2_, but a significant difference in the IC values of O and Si. However, the use of such different combinations of SCs does not modify the SS values. It is worth noting that SS values at the *M*(1,2,3,4) and *A*(*m*) sites obtained from model (*e*) were in excellent agreement with the chemical formula, while those from model (*f*) were only slightly smaller.

**Figure 3:**
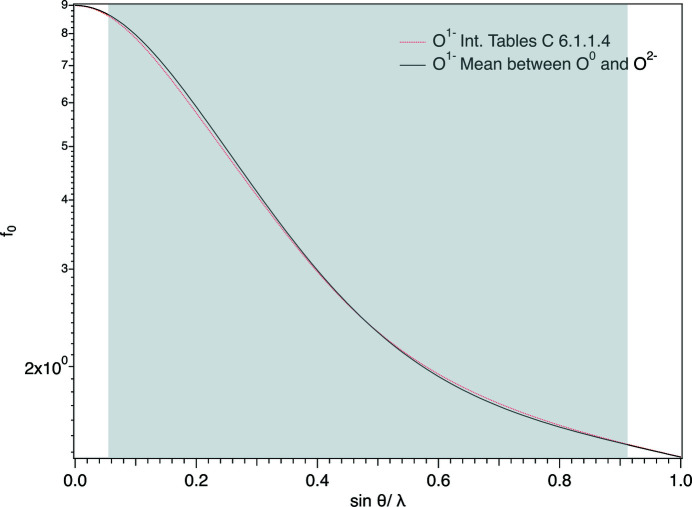
The sin θ/λ dependence of the scattering curve of O^1−^, as calculated from the coefficients listed in Table 6.1.1.4 of the *International Tables for Crystallography* (Brown *et al.*, 2006 ▸), and that obtained by combination of O^0^ and O^2 ^ scattering curves.

It is interesting to observe that the minor structural differences obtained by the optimization of O^2−^ versus O^0^, instead of O^2−^ versus O^1−^, are only apparently surprising. In fact, they can be explained by plotting *f*_0_ of O^1−^ (coefficients from the *International Tables for Crystallography*; Brown *et al.*, 2006 ▸) against *f*_0_ of O^2−^ and O^0^, giving large areas of no superposition (Fig. 3 ▸).

In particular, OOSC provided a total SS value at the *M*(1,2,3) sites of 61.05 (10) e^−^, in excellent agreement with 61.17 e^−^ from the chemical formula. Note that the refinement with NOC led to a significantly lower SS value of 59.99 (14) e^−^ for *M*(1,2,3) (Table 2 ▸). The increment in the SS value at the cation sites is coupled with a minor rise in the corresponding *U*_eq_, suggesting a redistribution of the electron density around the atoms. This redistribution leads, on the contrary, to the generalized decrease in *U*_eq_ of the various O and *T* sites. Nevertheless, such variations have to be considered marginal, as can be easily observed from the dispersion of the average values reported in the last column of Table 3 ▸.

As far as bond distances are concerned, differences among the various refinements are within experimental uncertainty (last column of Table 4 ▸). This confirms that the different types of SC mainly affect the redistribution of the electron density around the atoms without displacing the corresponding maxima.

With regard to the site populations, the ^*T*(1)^(Si_3.32_Al_0.68_) population, dictated by the chemical data, is in excellent agreement with the 0.60 a.p.f.u. Al, calculated from the equation of Oberti *et al.* (2007 ▸):

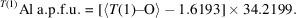

As previously indicated, *T*(2) was considered to host exclusively Si. However, throughout the various refinements we consistently observed a higher *U*_eq_ for *T*(2) than for *T*(1). This feature could possibly be an indication of the occurrence of minor ^*T*(2)^Al. By the evaluation of Fig. 11 of Oberti *et al.* (2007 ▸), a content of *ca* 0.1 ^*T*(2)^Al a.p.f.u. might be estimated from the observed 〈*T*(2)—O〉 of 1.634 Å. However, due to the extremely large data dispersion, Oberti *et al.* (2007 ▸) suggested that ‘*…we cannot presently provide a simple procedure to evaluate ^T(2)^Al based on structure refinement.*’. Therefore, *T*(2) was assumed to be fully occupied by Si.

The *M*(1,2,3) site populations were derived from the OOSC values according to the procedure of Vignaroli *et al.* (2014 ▸). This procedure consists of an iterative combined optimization at each *M*(1,2,3) site of both the SS values and the corresponding aggregate sizes of the constituent cations 〈*r^M^* 〉 (Table 5 ▸). In detail, Mg dominates over the three non-equivalent *M*(1,2,3) sites. Aluminium shows the site preference *M*(2) > *M*(3) >> *M*(1), in agreement with the occurrence of 〈*M*(3)—O〉 mean bond distance shorter than 〈*M*(1)—O〉 mean bond distance (Oberti *et al.*, 1995 ▸). In accordance with the site preference (Oberti *et al.*, 2007 ▸; Hawthorne *et al.*, 2012 ▸), Ti^4+^ is allocated at *M*(1), and V^3+^ and Cr^3+^ at *M*(2). Similarly, the *M*(4) site contains prevailing Ca plus minor amounts of Mg, Mn^2+^ and Fe^2+^, whereas *A*(*m*) hosts K and Na. Trace amounts of Na are also accommodated at the *A*(2) site, whose occurrence was detected from difference Fourier maps but not refined. The results of this iterative optimization procedure in terms of differences between the corresponding 〈*r^M^* 〉 values obtained from the observed bond distances and those calculated from the site populations are about 0.002 Å. Regarding the SS, the final site populations from OOSC SREF at the *M*(1,2,3,4) sites produced SS values smaller by 0.33% than those obtained from the chemical data. The larger discrepancy (*ca* 0.2 e^−^) obtained for *A*(*m*) may be ascribed to the simplified description adopted for the *A* site, which yields an increase in the anisotropic displacement parameters, with *U*_eq_ up to 0.0516 (15) Å^2^, to compensate for static disorder and in turn a reduction in the SS value.

Of particular interest is the refinement done with OOSC and fully ionized SCs for non-tetrahedrally coordinated cations. As reported above, the refinement of O^2−^ versus O^0^, instead of O^2−^ versus O^1−^, resulted in a marginal decrease in *wR*_2_ along with significant differences in the refined IC values for both O and Si. Moreover, while the IC values for O are very close to those observed in model (*e*), the IC values of Si are significantly higher, up to +1.730 for the refinement of O^2−^ versus O^0^. This value agrees with the range of +1 to +2 reported by Hawthorne *et al.* (1995 ▸) for Si in rock-forming minerals. Therefore, the use of fully ionized SCs for non-tetrahedrally coordinated cations affects the IC value of Si without affecting the SS values at the corresponding non-tetrahedrally coordinated sites, except for the slight increase in the SS value of *A*(*m*) mentioned above.

As a conclusion of this section, models (*f*) and (*g*) provide the best description of the present tremolite structure. Although these models are very similar, model (*f*) provides better statistics, as indicated by the *R* indices.

In order to extend the systematic analysis of the present sample, structural parameters including the optimized O and Si scattering curves (OOSC) of model (*f*) have been further investigated to different resolution limits of 2θ_max_: 81° (full data set), 75°, 70°, 65° and 60° (Table 6 ▸). The results show that regular trends occurr as follows.

(1) Progressive increases in the highest peak and deepest hole from the electron-density synthesis with coefficients (*F*_o_ − *F*_c_); the highest peak consistently corresponds to the *A*(2) sites, whereas the second peak corresponds to the *M*(4)′ site only for 2θ_max_ = 81°.

(2) Progressive decrease (from −1.378 to −1.288) and increase (from +0.776 to +0.887) in the ionic charges of O and Si, respectively.

(3) Progressive decrease in the SS values at the *M*(1,2,3,4) and *A*(*m*) sites. In detail, the sum of the SS at *M*(1,2,3) decreases from 61.28 to 61.05 e^−^ for 2θ_max_ = 60° and 81°, respectively.

(4) Progressive reduction (not listed in Table 6 ▸) of the number of largest correlation matrix elements larger than 0.5. In detail, for 2θ_max_ = 60°, moderate correlations occur between the principal axes of the ellipsoids describing the atom displacement parameters of the *M*(1,2,3) sites and the corresponding site occupancy factors. For 2θ_max_ = 81°, these correlations drop below 0.5.

(5) Progressive decrease in the atom displacement parameters of the *M* and *A* sites with increasing atom displacement parameters of the *T* and *O* sites.

(6) Progressive convergence of the bond-distance values towards those refined to 2θ_max_ = 81°. Differences from the refinement at 2θ_max_ = 60° are within 3σ, thus showing a dispersion larger than that arising from different choices of SCs.

### 4.3. Rietveld refinements   

Rietveld refinements of the data collected on the two capillaries provided fully reproducible structural data with differences within experimental uncertainty. Therefore, our discussion will focus on the results from one of the two samples only. Consistent with the type of experimental setup used, the spherical harmonics coefficients of the preferred orientation correction refined to very small values and they were remarkably constant for all refinements (Table 7 ▸). This behaviour is particularly important as it testifies that such correction, being constant, does not contribute to compensating for the inadequacy of the various structural models.

As a general remark, the use of fully ionized SCs for non-tetrahedrally coordinated cations consistently failed to reproduce the SS at the corresponding sites. Thus, we will not analyse in detail the corresponding structural results. Moreover, the smallest agreement indices are not associated with the closest description of the present tremolite structure with respect to SREF. Despite the complexity of the diffraction pattern and the number of refinable parameters, *R*_wp_ values of all refinements lay within the narrow range of 0.0290–0.0309. The lowest value was obtained by a combination of optimized SCs for both O and Si plus fully ionized SCs for non-tetrahedrally coordinated cations, that, as mentioned above, produce significantly overestimated SS at the various *M* sites. Therefore, it is clear that statistical indicators cannot be used as the only guide for selecting the best computational setup to perform such refinements. This is caused by the occurrence of several nearly equivalent (local) minima in the least-squares procedure. As in the case of SREF, we observed that refinement of O^2−^ versus O^0^ SCs instead of O^2−^ versus O^1−^ SCs produced a general, minor, improvement of the fit. In a further analogy with SREF, passing from NOC to O^2−^ SC has the effect of regularly increasing the SS at both *M* and *A* sites. Similar to SREF, individual and mean *M*(1,2,3)—O bond distances are almost unaffected (Table 8 ▸). It is worth noting that the use of increasingly ionized oxygen SCs generates, as a by-product, a regular increase in the absorption correction parameter. This effect is reasonable, as both SCs and absorption effects modulate the dependence of the calculated intensities on sinθ/λ. Therefore, it is crucial that the phenomenological representation adopted by the used Rietveld code might be able to reproduce as closely as possible the absorption effects caused by the sample, because small differences can affect the refined values of SS and displacement parameters to some extent.

The NOC refinement produced a structure in excellent agreement with SREF. The mean differences from OOSC SREF, expressed in the form of the number of standard uncertainties (σ), of the SS at the *M* sites (〈Δσ_*M* SS_〉) and of individual *T*—O and *M*—O bond distances (〈Δσ_BD_〉) were *ca* 2.7 and 3.3, respectively. The total SS at *M*(1,2,3) refined to 60.5 (2) e^−^, 0.5 e^−^ less than that obtained from SREF and *ca* 0.7 e^−^ less than that calculated from site partitioning using the EMPA data. This behaviour was previously reported and quantified by Ballirano *et al.* (2017 ▸) and Ballirano & Pacella (2020 ▸). In particular, SS at *M*(2) was consistently found to be moderately underestimated. This is exactly the same result as obtained in the present refinement that shows an excellent fit with the EPMA data of the SS at *M*(1) and *M*(3).

The SS at *A*(*m*) is higher than that obtained from both SREF and EMPA, but it represents the only significant discrepancy between the two structural data sets. Note that this behaviour is consistent with that observed in reference Rietveld refinements performed by our research group. In detail, we found minor electron density at *A* sites even in the case of virtually alkali-free tremolite samples, suggesting that this overestimation is actually an artefact. This effect could possibly be amplified, as observed in SREF, by the generalized use of simplified structural modelling of those sites [*A*(*m*) or *A*(2/*m*) and fixed/isotropic displacement parameters].

As far as bond distances are concerned, the mean *T*—O and *M*—O bond distances were within ± 0.009 Å of the corresponding value from OOSC SREF. The 〈〈*r* ^*M*(1,2,3)^〉〉 of 0.711 Å from Rietveld refinement is slightly larger than the value of 0.706 Å from OOSC SREF. We deliberately avoid any discussion of the behaviour of the individual and mean *A*(*m*)—O bond distances owing to the significantly higher associated standard uncertainties compared with those of the *T*—O and *M*—O bond distances.

The use of increasingly reduced oxygen SCs produces a very minor increase in *R*_wp_ and marginally alters the mean bond distances as 〈Δσ_BD_〉 is always close to 3.4. In the case of the refinement using the O^1−^ SC, the results indicate a structure very close to that of NOC, albeit coupled to slightly higher agreement indices. The OOC refinement produced results intermediate between those obtained with O^2−^ and O^1−^ SCs. The 〈〈*r* ^*M*(1,2,3)^〉〉 reaches a value of 0.713 Å in the case of choosing OOC. In contrast, 〈Δσ_*M* SS_〉 increases progressively. This is predominantly due to the large rise in SS at *M*(4). Moreover, SS at *A*(*m*) increases as well.

The OOSC refinement produced results similar to those of NOC. The value of *R*_wp_ was marginally smaller at *M*(1,2,3), as was SS that reached a value of 60.3 (2) e^−^. In contrast, both *M*(4) and *A*(*m*) refined to higher SS values. Where OOSC performed significantly worse than NOC was in the case of 〈〈*r* ^*M*(1,2,3)^〉〉 that was calculated to 0.716 Å, a significantly higher value than 0.711 Å from NOC and, especially, than 0.706 Å from OOSC SREF.

In the case of choosing fully oxidized SCs for non-tetrahedrally coordinated cations, as mentioned at the beginning of this section, they produced a very large overestimation of SS at *M* sites, whereas a better agreement than neutral ones was observed at *A*(*m*). The difference in the mean bond distances with respect to SREF 〈Δσ_BD_〉 was slightly higher that that observed in the case of the use of neutral SCs from non-tetrahedral cations, being close to 4.

Therefore, according to the present results, the use of NOC produces the best agreement with SREF structural data. However, a few regular behaviours were observed, confirming the findings of previous Rietveld refinements of fibrous amphiboles with the same experimental setup:

(1) Total SS at the *M*(1,2,3) sites is consistently underestimated from Rietveld refinements and such underestimation is almost completely taken up by *M*(2). The underestimation is proportional to the total SS at the *M*(1,2,3) sites (Ballirano *et al.*, 2017 ▸);

(2) The ‘missing’ SS seems to be (partly) re-allocated at the *A* site(s);

(3) Mean *T*— and *M*—O bond distances are expected to be within ± 0.01 Å from SREF (in the present case 0.003–0.009 Å);

(4) Discrepancies of individual bond distances (not reported) are obviously greater than those of the mean values and they could possibly be reduced by imposing restraints [maximum discrepancy in the present case 0.027 Å for *T*(2)—O(2)].

The effects of using different types of SCs for amphiboles are significantly different than for simpler structures such as spinel (Ballirano, 2003 ▸). In that case, the use of optimized SCs for oxygen (in particular O^1.7−^, in close agreement with SREF) produced better SS, and better displacement parameter modelling and agreement indices as well. This is possibly due to several concurrent reasons, such as the very limited superposition of reflections caused by the high symmetry and smaller absorption, which is generally better modelled than higher values, and a slightly extended 2θ range (Ballirano, 2014 ▸).

## 5. Conclusions   

In the present study, we have shown that the interpretation of the electron density of the tremolite structure is model-dependent. As a valid constraint for the correct description of the model, we assumed that SS values from SREF should be as close as possible to those from EMPA. This assumption was best satisfied by using partially ionized SCs for O and Si, and neutral SCs for the other atoms, which also led to the best fit to the diffraction data. In this regard, it should be noted that the SCs used in SREF are calculated for isolated atoms or ions (*e.g.* Brown *et al.*, 2006 ▸), thus their optimization as neutral versus ionized SCs should accommodate the effective cation–anion bonding environment occurring in the real local structure. Hence, a structural model can be considered physically valid when its site occupancies are in agreement with the composition obtained by chemical analysis, which provides charge-balanced mineral formulae. Regarding the present tremolite, for example, the sum of SS values at the *M*(1,2,3,4) and *A*(*m*) sites obtained from EMPA (105.07 e^−^) is closer to that obtained from the SREF model with partially ionized and neutral SCs (104.63 e^−^) than to that obtained from the SREF model with all neutral SCs (102.02 e^−^). Moreover, the experimental data should include, whenever possible, high-angle reflections (2θ > 60°) because they contribute to reducing correlation(s) and provide an improved description of the crystal structure.

As far as Rietveld refinements are concerned, NOC has produced structural results in excellent agreement with single-crystal SREF. However, due to the (relative) complexity of the diffraction pattern and the corresponding high number of freely refinable parameters, refinements performed with different combinations of SCs produced results almost indistinguishable from a statistical viewpoint. This is probably due to the correlation between absorption effects and the shapes of the various SCs, mutually compensating each other. Therefore, under the selected experimental conditions it is recommended to use NOC, which produced the closest structural representation of the tremolite sample compared with SREF in terms of both SS and bond distances. Those computational conditions led to an underestimation of the total SS at the *M*(1,2,3) sites, predominantly taken up by *M*(2), and to *T*(1,2)— and *M*(1,2,3,4)—O mean bond distances within ± 0.01 Å of the SREF values. However, before generalizing the obtained results to the analysis of fibrous amphiboles, some caution should be exercised. In fact, the analysed sample consisted of a chemically homogeneous fragment of a large gem-quality crystal. In contrast, large compositional variation is not uncommon in amphiboles when analysing fibres from a certain location. In fact, the term ‘suite’ has often been used to describe the large chemical variability observed at a few ‘classic’ locations, in some cases embracing as many as three amphibole species [*e.g.* Libby, Montana, USA (Wylie & Verkouteren, 2000 ▸; Gunter *et al.*, 2003 ▸; Meeker *et al.*, 2003 ▸) and Biancavilla, Sicily, Italy (Andreozzi *et al.*, 2009 ▸)]. This feature has also been observed in the case of erionite, a fibrous zeolite posing even greater problems than asbestos amphiboles as far as human health is concerned [Rome, Oregon, USA (Ballirano *et al.*, 2009 ▸; Ballirano & Cametti, 2015 ▸; Pacella *et al.*, 2017 ▸)]. Under such premises, it is clear that chemical variability represents a limiting factor more severe than the limitations imposed by the Rietveld method itself. In this case it is recommended to report a measure of the strain-related peak broadening (Ballirano & Sadun, 2009 ▸) in order to provide its rough estimate, keeping in mind that strain broadening could also be due to other concurrent reasons such as fibre curling or bending.

What has been shown in this study is that to solve the site occupancy issues of any crystalline substance, the structure refinement results should not be limited to a simple description of the crystal geometry (bond distances, angles *etc.*) but should go beyond that, providing refined SS values consistent with the chemical data. In this regard, it should be noted that the refined SS value can be interpreted in terms of atomic constituents *only* if the chemical information is incorporated into the interpretation procedure. The resulting crystal-chemical model (summarized by the corresponding structural formula) can hence be evaluated, for example, as the best match between the number of electrons obtained from SREF and EMPA data. Recently, Hawthorne *et al.* (2021 ▸) used a similar approach to refine the SS values for a new ortho­rhombic amphibole (ferropapikeite) in order to evaluate the most accurate site populations. The recommendations they provided can also be generalized to other complex minerals, as shown for example by the improved descriptions of the crystal structures of spinel (Andreozzi *et al.*, 2000 ▸), Tutton’s salt (Ballirano *et al.*, 2007 ▸), kornerupine (Cooper *et al.*, 2009 ▸), tourmaline (Lussier *et al.*, 2011 ▸) and pyrochlore (Hålenius & Bosi, 2013 ▸).

## Supplementary Material

Crystal structure: contains datablock(s) tremolite, powder. DOI: 10.1107/S2052520621004844/yh5012sup1.cif

Structure factors: contains datablock(s) tremolite. DOI: 10.1107/S2052520621004844/yh5012tremolitesup2.hkl

Rietveld powder data: contains datablock(s) powder. DOI: 10.1107/S2052520621004844/yh5012powdersup3.rtv

CCDC references: 2082220, 2082221

## Figures and Tables

**Table 1:** Chemical composition for the tremolite studied Uncertainties for oxides and fluorine (in brackets) are the standard deviation of 15 EMPA spots across the crystal used for SREF study. Number of ions normalized to 24 anions.

Oxides	Wt%	Range	Ions	Atoms per formula unit
SiO_2_	53.07 (34)	52.39–53.61	Si	7.320
TiO_2_	0.28 (4)	0.21–0.34	Ti	0.030
Al_2_O_3_	6.29 (16)	5.94–6.56	Al	1.023
V_2_O_3_	0.36 (3)	0.31–0.43	V^3+^	0.040
Cr_2_O_3_	0.07 (2)	0.03–0.10	Cr	0.007
FeO	0.06 (3)	0.0–0.11	Fe^2+^	0.007
MnO	0.14 (4)	0.08–0.21	Mn^2+^	0.016
MgO	22.43 (18)	22.13–22.66	Mg	4.612
CaO	13.17 (17)	12.78–13.44	Ca	1.945
Na_2_O	0.69 (3)	0.64–0.74	Na	0.185
K_2_O	0.60 (4)	0.49–0.64	K	0.105
F	0.76 (14)	0.59–0.98	F	0.331
H_2_O† ^a^	1.82		OH	1.609
O=F	−0.32		O	0.060
Total	99.41			

† Calculated by assuming ^W^(O + OH + F) = 2.000 a.p.f.u.

**Table 2:** Statistical indicators, highest peak and deepest hole (in e^−^ Å^−3^) from the electron-density synthesis with coefficients (*F*_o_ − *F*_c_), maximum and average value of error/s.u. and average resolution (in Å) of the most disagreeable reflections (average values calculated over the ten most disagreeable ones), ionic charges (IC) for O and Si [calculated from the refined occupancy of ionized versus ionized or neutral SCs for oxygen (O^2−^ versus O^1−^ or O^0^), and ionized versus neutral species for cations at *T* (Si^4+^ versus Si^0^) through the FVAR instruction in the *SHELXL-2013* program], and site scattering (SS, in e^−^) of tremolite as a function of different combinations (ABC)^*x*^ of scattering curves (SCs) from SREF (ABC)^0^ and (ABC)^*n*+^ refer to neutral and fully ionized SCs, for Mg^0^/Mg^2+^ at *M*(1,2,3), Ca^0^/Ca^2+^ at *M*(4) and K^0^/K^1+^ at *A*(*m*). Weighting scheme defined as *w* = 1/[σ^2^(*F*_o_^2^) + (*aP*)^2^ + *bP*], where *P* is [2*F*_c_^2^ + Max(*F*_o_^2^, 0]/3 (Sheldrick, 2015 ▸). ‘Vs’ is an abbreviation for versus. GooF stands for goodness of fit.

		(*a*)	(*b*)	(*c*)	(*d*)	(*e*)		(*f*)		(*g*)	
		O^0^	O^0^	O^1−^	O^2−^	O^1−^ vs O^2−^	O^0^ vs O^2−^	O^1−^ vs O^2−^	O^0^ vs O^2−^	O^1−^ vs O^2−^	O^0^ vs O^2−^
		Si^0^	Si^0^	Si^0^	Si^0^	Si^0^	Si^0^	Si^0^ vs Si^4+^	Si^0^ vs Si^4+^	Si^0^ vs Si^4+^	Si^0^ vs Si^4+^
		(ABC)^0^	(ABC)^*n*+^	(ABC)^0^	(ABC)^0^	(ABC)^0^	(ABC)^0^	(ABC)^0^	(ABC)^0^	(ABC)^*n*+^	(ABC)^*n*+^
*wR*_2_		0.0458	0.0517	0.0417	0.0373	0.0347	0.0337	0.0315	0.0314	0.0332	0.0330
*R*_1_ for *I* > 2σ(*I*)		0.0150	0.0152	0.0143	0.0144	0.0135	0.0134	0.0133	0.0132	0.0136	0.0136
*R*_1_ for all reflections		0.0164	0.0165	0.0157	0.0158	0.0149	0.0148	0.0147	0.0147	0.0150	0.0150
GooF		1.190	1.163	1.143	1.135	1.149	1.143	1.119	1.116	1.146	1.139
Weighting scheme	*a*	0.0183	0.0244	0.0179	0.0141	0.0141	0.0135	0.0118	0.0119	0.0127	0.0130
	*b*	0.5579	0.5615	0.4552	0.467	0.3013	0.2953	0.3078	0.3016	0.3014	0.2928
Highest peak		0.59	0.63	0.63	0.71	0.68	0.67	0.65	0.64	0.65	0.65
Deepest hole		−0.34	−0.34	−0.29	−0.32	−0.31	−0.29	−0.34	−0.32	−0.36	−0.34
Most disagreeable reflections	Maximum error/s.u.	17.17	17.77	13.92	13.61	14.62	13.53	5.79	5.46	7.42	7.41
	Average error/s.u.	9.16	9.62	8.42	6.76	5.71	5.40	4.74	4.51	5.19	5.03
	Average resolution	2.51	2.51	2.42	2.97	2.55	2.55	2.26	2.30	2.15	2.21
IC O		0	0	0	0	−1.568	−1.270	−1.560	−1.288	−1.566	−1.301
IC Si		0	0	0	0	0	0	+1.001	+0.887	+1.841	+1.730
*M*(1) SS		23.81 (5)	23.82 (5)	23.93 (5)	24.56 (4)	24.31 (4)	24.30 (4)	24.24 (4)	24.25 (4)	24.23 (4)	24.24 (4)
*M*(2) SS		24.34 (5)	24.35 (5)	24.44 (4)	25.07 (4)	24.82 (4)	24.81 (4)	24.72 (4)	24.74 (4)	24.70 (4)	24.72 (4)
*M*(3) SS		11.84 (4)	11.85 (4)	11.91 (3)	12.21 (3)	12.10 (3)	12.09 (3)	12.05 (3)	12.06 (3)	12.06 (3)	12.06 (3)
Σ_C_ SS		59.99 (14)	60.02 (15)	60.28 (12)	61.84 (12)	61.22 (11)	61.21 (10)	61.01 (10)	61.05 (10)	60.98 (10)	61.02 (10)
*M*(4) SS		39.26 (5)	39.33 (6)	39.43 (5)	40.20 (5)	39.88 (4)	39.87 (4)	39.74 (4)	39.76 (4)	39.77 (4)	39.79 (4)
*A*(*m*) SS		3.77 (7)	3.90 (7)	3.86 (6)	4.04 (6)	3.95 (5)	3.93 (5)	3.83 (5)	3.82 (5)	3.93 (5)	3.92 (5)

**Table 3:** Displacement parameters *U*_eq_ [*U*_iso_ for H(3)] (all in Å^2^) of tremolite as a function of different combinations (ABC)^*x*^ of SCs from SREF ‘vs’ is an abbreviation of versus.

	(*a*)	(*b*)	(*c*)	(*d*)	(*e*)		(*f*)		(*g*)		
	O^0^	O^0^	O^1−^	O^2−^	O^1−^ vs O^2−^	O^0^ vs O^2−^	O^1−^ vs O^2−^	O^0^ vs O^2−^	O^1−^ vs O^2−^	O^0^ vs O^2−^	
	Si^0^	Si^0^	Si^0^	Si^0^	Si^0^	Si^0^	Si^0^ vs Si^4+^	Si^0^ vs Si^4+^	Si^0^ vs Si^4+^	Si^0^ vs Si^4+^	
	(ABC)^0^	(ABC)^*n*+^	(ABC)^0^	(ABC)^0^	(ABC)^0^	(ABC)^0^	(ABC)^0^	(ABC)^0^	(ABC)^*n*+^	(ABC)^*n*+^	Average
*M*(1)	0.00544 (8)	0.00537 (8)	0.00556 (7)	0.00602 (7)	0.00586 (6)	0.00584 (6)	0.00581 (6)	0.00580 (6)	0.00573 (6)	0.00573 (6)	0.0057 (2)
*M*(2)	0.00438 (7)	0.00431 (8)	0.00449 (7)	0.00492 (6)	0.00476 (6)	0.00475 (6)	0.00469 (5)	0.00470 (6)	0.00460 (6)	0.00460 (6)	0.00462 (19)
*M*(3)	0.00502 (11)	0.00496 (11)	0.00516 (10)	0.00559 (9)	0.00544 (8)	0.00542 (8)	0.00538 (8)	0.00537 (8)	0.00533 (8)	0.00532 (8)	0.0053 (2)
*M*(4)	0.00837 (4)	0.00836 (5)	0.00847 (4)	0.00874 (4)	0.00865 (3)	0.00864 (3)	0.00859 (3)	0.00858 (3)	0.00858 (3)	0.00857 (3)	0.00856 (12)
*T*(1)	0.00470 (4)	0.00466 (4)	0.00469 (3)	0.00442 (3)	0.00454 (3)	0.00453 (3)	0.00453 (3)	0.00451 (3)	0.00446 (3)	0.00445 (3)	0.00455 (10)
*T*(2)	0.00506 (4)	0.00500 (4)	0.00504 (3)	0.00478 (3)	0.00490 (3)	0.00489 (3)	0.00488 (3)	0.00486 (3)	0.00481 (3)	0.00479 (3)	0.00490 (10)
O(1)	0.00742 (7)	0.00735 (6)	0.00727 (6)	0.00667 (6)	0.00694 (5)	0.00696 (5)	0.00698 (5)	0.00698 (5)	0.00695 (5)	0.00695 (5)	0.0070 (2)
O(2)	0.00708 (7)	0.00704 (7)	0.00693 (6)	0.00630 (6)	0.00658 (5)	0.00660 (5)	0.00662 (5)	0.00662 (5)	0.00659 (5)	0.00659 (5)	0.0067 (2)
O(3)	0.00842 (9)	0.00837 (10)	0.00830 (8)	0.00774 (8)	0.00799 (7)	0.00800 (7)	0.00803 (7)	0.00803 (7)	0.00801 (7)	0.00800 (7)	0.0081 (2)
H(3)	0.0101	0.0100	0.0100	0.0093	0.0096	0.0096	0.0096	0.0096	0.0096	0.0096	0.0097 (3)
O(4)	0.00919 (7)	0.00912 (7)	0.00903 (6)	0.00845 (6)	0.00870 (6)	0.00872 (5)	0.00875 (5)	0.00875 (5)	0.00872 (5)	0.00873 (5)	0.0088 (2)
O(5)	0.00978 (7)	0.00971 (8)	0.00962 (7)	0.00905 (6)	0.00931 (6)	0.00933 (6)	0.00935 (6)	0.00936 (6)	0.00933 (6)	0.00933 (6)	0.0094 (2)
O(6)	0.00909 (7)	0.00903 (8)	0.00894 (7)	0.00837 (6)	0.00862 (6)	0.00864 (6)	0.00867 (5)	0.00867 (5)	0.00864 (5)	0.00864 (5)	0.0087 (2)
O(7)	0.01112 (10)	0.01108 (11)	0.01095 (9)	0.01033 (9)	0.01060 (8)	0.01062 (8)	0.01063 (8)	0.01064 (8)	0.01061 (8)	0.01062 (8)	0.0107 (3)
*A*(*m*)	0.051 (2)	0.053 (2)	0.053 (2)	0.0540 (9)	0.0536 (17)	0.0533 (16)	0.0518 (16)	0.0516 (15)	0.0532 (16)	0.0531 (16)	0.0528 (9)

**Table 4:** Relevant bond distances (in Å) of tremolite as a function of the different choice (ABC)^*x*^ of the SCs of oxygen from SREF ‘vs’ is an abbreviation of versus

		(*a*)	(*b*)	(*c*)	(*d*)	(*e*)		(*f*)		(*g*)		
		O^0^	O^0^	O^1−^	O^2−^	O^1−^ vs O^2−^	O^0^ vs O^2−^	O^1−^ vs O^2−^	O^0^ vs O^2−^	O^0^ vs O^2−^	O^1−^ vs O^2−^	
		Si^0^	Si^0^	Si^0^	Si^0^	Si^0^	Si^0^	Si^0^ vs Si^4+^	Si^0^ vs Si^4+^	Si^0^ vs Si^4+^	Si^0^ vs Si^4+^	
		(ABC)^0^	(ABC)^*n*+^	(ABC)^0^	(ABC)^0^	(ABC)^0^	(ABC)^0^	(ABC)^0^	(ABC)^0^	(ABC)^*n*+^	(ABC)^*n*+^	Average
*T*(1)	—O(7)	1.6300 (3)	1.6301 (3)	1.6300 (3)	1.6299 (3)	1.6299 (2)	1.6299 (2)	1.6298 (2)	1.6298 (2)	1.6298 (2)	1.6297 (2)	1.6299 (1)
	—O(6)	1.6465 (5)	1.6466 (5)	1.6465 (4)	1.6465 (4)	1.6465 (4)	1.6465 (4)	1.6464 (3)	1.6464 (3)	1.6464 (3)	1.6464 (3)	1.6465 (1)
	—O(1)	1.6218 (4)	1.6220 (5)	1.6221 (4)	1.6224 (4)	1.6224 (3)	1.6223 (3)	1.6221 (3)	1.6220 (3)	1.6221 (3)	1.6222 (3)	1.6221 (2)
	—O(5)	1.6493 (5)	1.6494 (5)	1.6493 (4)	1.6490 (4)	1.6491 (4)	1.6491 (4)	1.6492 (3)	1.6492 (3)	1.6494 (4)	1.6494 (4)	1.6492 (1)
〈*T*(1)—O〉		1.6369	1.6370	1.6370	1.6369	1.6370	1.6370	1.6369	1.6369	1.6369	1.6369	1.6369 (1)
												
*T*(2)	—O(4)	1.5930 (5)	1.5933 (5)	1.5930 (4)	1.5928 (4)	1.5929 (4)	1.5929 (4)	1.5927 (4)	1.5927 (3)	1.5928 (4)	1.5928 (4)	1.5929 (2)
	—O(5)	1.6527 (4)	1.6528 (5)	1.6527 (4)	1.6529 (4)	1.6528 (4)	1.6528 (3)	1.6526 (3)	1.6526 (3)	1.6526 (3)	1.6525 (3)	1.6527 (1)
	—O(2)	1.6206 (4)	1.6208 (5)	1.6207 (4)	1.6208 (4)	1.6208 (3)	1.6208 (3)	1.6206 (3)	1.6205 (3)	1.6206 (3)	1.6206 (3)	1.6207 (1)
	—O(6)	1.6691 (5)	1.6692 (5)	1.6691 (4)	1.6688 (4)	1.6689 (4)	1.6689 (4)	1.6688 (3)	1.6688 (3)	1.6690 (3)	1.6690 (4)	1.6690 (1)
〈*T*(2)—O〉		1.6339	1.6340	1.6339	1.6338	1.6339	1.6339	1.6337	1.6337	1.6338	1.6337	1.6338 (1)
												
*M*(1)	—O(3) ×2	2.0830 (5)	2.0828 (5)	2.0829 (4)	2.0828 (4)	2.0828 (4)	2.0828 (3)	2.0829 (3)	2.0829 (3)	2.0828 (3)	2.0828 (3)	2.0828 (1)
	—O(1) ×2	2.0551 (4)	2.0550 (5)	2.0549 (4)	2.0543 (4)	2.0545 (3)	2.0546 (3)	2.0546 (3)	2.0546 (3)	2.0545 (3)	2.0545 (3)	2.0547 (3)
	—O(2) ×2	2.0809 (5)	2.0808 (5)	2.0809 (4)	2.0814 (4)	2.0811 (4)	2.0811 (4)	2.0813 (4)	2.0813 (4)	2.0813 (4)	2.0813 (3)	2.0811 (2)
〈*M*(1)—O〉		2.0730	2.0729	2.0729	2.0728	2.0728	2.0728	2.0729	2.0729	2.0729	2.0729	2.0729 (1)
												
*M*(2)	—O(4) ×2	1.9963 (5)	1.9960 (5)	1.9962 (4)	1.9965 (4)	1.9964 (4)	1.9964 (4)	1.9966 (4)	1.9967 (4)	1.9964 (4)	1.9964 (4)	1.9964 (2)
	—O(2) ×2	2.0746 (4)	2.0745 (5)	2.0744 (4)	2.0737 (4)	2.0740 (3)	2.0740 (3)	2.0741 (3)	2.0741 (3)	2.0739 (3)	2.0739 (3)	2.0741 (3)
	—O(1) ×2	2.1102 (5)	2.1103 (5)	2.1101 (4)	2.1102 (4)	2.1100 (4)	2.1101 (4)	2.1102 (4)	2.1102 (4)	2.1103 (4)	2.1103 (4)	2.1102 (1)
〈*M*(2)—O〉		2.0604	2.0603	2.0602	2.0601	2.0601	2.0602	2.0603	2.0603	2.0602	2.0602	2.0602 (1)
												
*M*(3)	—O(1) ×4	2.0666 (4)	2.0664 (4)	2.0665 (4)	2.0665 (4)	2.0665 (3)	2.0666 (3)	2.0666 (3)	2.0666 (3)	2.0665 (3)	2.0665 (3)	2.0665 (1)
	—O(3) ×2	2.0580 (5)	2.0579 (7)	2.0578 (6)	2.0576 (5)	2.0576 (5)	2.0576 (5)	2.0577 (5)	2.0578 (5)	2.0577 (5)	2.0577 (5)	2.0577 (1)
〈*M*(3)—O〉		2.0637	2.0636	2.0636	2.0635	2.0635	2.0633	2.0636	2.0637	2.0636	2.0636	2.0636 (1)
												
〈〈*M*(1,2,3)—O〉〉		2.0657	2.0656	2.0656	2.0655	2.0655	2.0655	2.0656	2.0656	2.0655	2.0655	2.0656 (1)
〈〈*r* ^*M*(1,2,3)^〉〉†		0.706	0.706	0.706	0.706	0.706	0.706	0.706	0.706	0.706	0.706	0.706 (0)
												
*M*(4)	—O(4) ×2	2.3345 (5)	2.3344 (5)	2.3344 (4)	2.3342 (4)	2.3342 (4)	2.3342 (3)	2.3344 (3)	2.3344 (3)	2.3344 (3)	2.3344 (3)	2.3344 (3)
	—O(2) ×2	2.4078 (5)	2.4077 (5)	2.4077 (4)	2.4074 (4)	2.4076 (4)	2.4076 (3)	2.4077 (3)	2.4077 (3)	2.4076 (3)	2.4076 (3)	2.4076 (3)
	—O(6) ×2	2.5380 (5)	2.5378 (5)	2.5379 (4)	2.5385 (4)	2.5384 (4)	2.5385 (4)	2.5386 (4)	2.5386 (4)	2.5386 (4)	2.5385 (4)	2.5386 (4)
	—O(5) ×2	2.7035 (5)	2.7033 (5)	2.7035 (4)	2.7035 (4)	2.7036 (4)	2.7036 (4)	2.7037 (4)	2.7037 (4)	2.7034 (4)	2.7035 (4)	2.7034 (4)
〈*M*(4)—O〉		2.4960	2.4958	2.4959	2.4959	2.4960	2.4960	2.4961	2.4961	2.4960	2.4960	2.4960 (1)
												
*A*(*m*)	—O(7)	2.489 (6)	2.489 (7)	2.488 (6)	2.486 (6)	2.487 (5)	2.487 (5)	2.487 (5)	2.487 (5)	2.488 (5)	2.487 (5)	2.488 (1)
	—O(7)	2.531 (7)	2.530 (7)	2.531 (6)	2.532 (9)	2.532 (5)	2.532 (5)	2.532 (5)	2.532 (5)	2.532 (5)	2.532 (5)	2.532 (1)
	—O(6) ×2	2.881 (4)	2.880 (4)	2.880 (4)	2.883 (3)	2.882 (3)	2.882 (3)	2.883 (3)	2.883 (3)	2.882 (3)	2.882 (3)	2.882 (1)
	—O(5) ×2	2.971 (4)	2.971 (4)	2.971 (3)	2.973 (3)	2.972 (3)	2.971 (3)	2.973 (3)	2.973 (3)	2.973 (3)	2.973 (3)	2.972 (1)
	—O(5) ×2	3.075 (4)	3.075 (4)	3.075 (3)	3.073 (3)	3.074 (3)	3.074 (3)	3.074 (3)	3.074 (3)	3.074 (3)	3.074 (3)	3.074 (1)
〈*A*(*m*)—O〉		2.859	2.859	2.859	2.860	2.859	2.859	2.860	2.860	2.860	2.860	2.860 (1)

† Calculated as in Table 7 of Hawthorne & Oberti (2007 ▸).

**Table 5:** SS values at the *T*(1,2), *M*(1,2,3,4) and *A*(*m*) sites calculated from SREF, the empirical structural formula and Rietveld refinement for the tremolite studied Optimized site populations are derived from the aggregate sizes of the constituent cations 〈*r^M^* 〉 and the SS.

	SS (e^−^)			Site population optimized from 〈*r^M^* 〉 and SS		
Site	SREF	From empirical structural formula	XRPD	SREF	〈*r^M^* 〉 from bond distances	〈*r^M^* 〉 optimized
*T*(1)	55.32	55.32	55.60 (0)	[Si_3.32_Al_0.68_], [Si_3.40_Al_0.60_]†		
*T*(2)	56.00	56.00	56 (0)	Si_4.00_		
Σ_*T*(1)+*T*(2)_	111.32	111.32	111.60			
*M*(1)	24.25 (4)	24.33	24.17 (8)	[Mg_1.940_Al_0.030_Ti_0.030_]	0.713	0.715
*M*(2)	24.74 (4)	24.74	24.34 (8)	[Mg_1.740_Al_0.213_V_0.040_Cr_0.007_]	0.700	0.698
*M*(3)	12.06 (3)	12.10	12.03 (6)	[Mg_0.900_Al_0.100_]	0.704	0.702
Σ_*M*(1)+*M*(2)+*M*(3)_	61.05 (10)	61.17	60.5 (2)	[Mg_4.580_Al_0.343_Ti_0.030_V_0.040_Cr_0.007_]	0.706	0.706
*M*(4)	39.76 (4)	39.87	39.68 (9)	Ca_1.945_Fe_0.007_Mn_0.016_Mg_0.032_		
*A*(*m*)	3.82 (5)	4.03	5.03 (7)	Na_0.185_K_0.105_		

† Si and Al populations at *T*(1) are according to the equation of Oberti *et al.* (2007 ▸).

**Table 6:** Statistical indicators, highest peak and deepest hole (in e^−^ Å^−3^) from the electron-density synthesis with coefficients (*F*_o_ − *F*_c_), ionic charges (IC) for O and Si, and SS (in e^−^) of tremolite as a function of 2θ_max_ (in °) from SREF with optimized oxygen and silicon SCs (OOSC) GooF stands for goodness of fit.

2θ_max_ (°)	60	65	70	75	81 (full data set)
sinθ/λ_max_ (Å^−1^)	0.7035	0.7560	0.8070	0.8565	0.9100
Unique reflections	1366	1682	2049	2440	2920
*R*_int_	0.0157	0.0160	0.0164	0.0167	0.0171
*R*_sigma_	0.0073	0.0076	0.0081	0.0087	0.0095
*wR*_2_	0.0284	0.0286	0.0289	0.0299	0.0314
*R*_1_ for *I* > 2σ(*I*)	0.0112	0.0113	0.0118	0.0122	0.0132
*R*_1_ for all reflections	0.0117	0.0119	0.0126	0.0132	0.0147
GooF	1.171	1.133	1.122	1.133	1.116
Highest peak	0.36	0.44	0.55	0.58	0.64
Deepest hole	−0.21	−0.24	−0.24	−0.29	−0.32
IC O	−1.378	−1.352	−1.330	−1.312	−1.288
IC Si	+0.776	+0.804	+0.836	+0.860	+0.887
*M*(1) SS	24.35 (6)	24.30 (5)	24.29 (4)	24.27 (4)	24.25 (4)
*M*(2) SS	24.82 (5)	24.79 (5)	24.76 (4)	24.76 (4)	24.74 (4)
*M*(3) SS	12.10 (4)	12.08 (3)	12.07 (3)	12.07 (3)	12.06 (3)
Σ_C_ SS	61.28 (15)	61.17 (13)	61.12 (12)	61.09 (11)	61.05 (10)
*M*(4) SS	40.04 (6)	39.97 (5)	39.88 (5)	39.83 (4)	39.76 (4)
*A*(*m*) SS	4.00 (6)	3.96 (6)	3.90 (5)	3.86 (5)	3.82 (5)

**Table 8:** Relevant parameters of the Rietveld refinements of tremolite as a function of a selection of different combinations (ABC)^*x*^ of SC The listed data are: statistical indicators as defined by Young (1993 ▸), absorption correction parameter (Sabine *et al.*, 1998 ▸), *M*-sites site scattering (in e^−^), mean *T*— and *M*—O bond distances (in Å), mean difference from the SREF optimized refinement of *M*-sites site scattering (〈Δσ_*M*SS_〉), and individual *T*— and *M*—O bond distances (〈Δσ_BD_〉), expressed as the number of σ. DWd denotes Durbin–Watson "d" statistic, GooF stands for goodness of fit and ‘vs’ is an abbreviation of versus.

	O^0^	O^1−^	O^2−^	O^1−^ vs O^2−^	O^0^ vs O^2−^	O^1−^ vs O^2−^	O^0^ vs O^2−^	O^0^	O^1−^	O^2−^	O^0^ vs O^2−^	
	Si^0^	Si^0^	Si^0^	Si^0^	Si^0^	Si^0^ vs Si^4+^	Si^0^ vs Si^4+^	Si^0^	Si^0^	Si^0^	Si^0^ vs Si^4+^	
	(ABC)^0^	(ABC)^0^	(ABC)^0^	(ABC)^0^	(ABC)^0^	(ABC)^0^	(ABC)^0^	(ABC)^*n*+^	(ABC)^*n*+^	(ABC)^*n*+^	(ABC)^*n*+^	SREF
*R*_wp_	0.0295	0.0297	0.0304	0.0302	0.0298	0.0295	0.0291	0.0306	0.0308	0.0309	0.0290	
*R*_p_	0.0220	0.0223	0.0229	0.0228	0.0224	0.0222	0.0219	0.0225	0.0227	0.0230	0.0216	
DWd	0.262	0.256	0.245	0.248	0.255	0.259	0.265	0.260	0.253	0.249	0.279	
GooF	3.779	3.803	3.885	3.863	3.829	3.758	3.725	3.917	3.935	3.954	3.707	
*R*_Bragg_	0.0146	0.0148	0.0156	0.0154	0.0148	0.0147	0.0144	0.0155	0.0157	0.0158	0.0140	
Absorption correction	2.365 (9)	2.430 (9)	2.510 (9)	2.476 (9)	2.461 (9)	2.412 (9)	2.426 (9)	2.317 (9)	2.382 (9)	2.455 (10)	2.347 (9)	
*M*(1) SS	24.17 (8)	24.36 (8)	24.64 (9)	24.52 (8)	24.50 (8)	24.10 (8)	24.16 (8)	25.24 (9)	25.38 (9)	25.63 (9)	25.04 (9)	24.25 (4)
*M*(2) SS	24.34 (8)	24.43 (8)	24.61 (9)	24.60 (9)	24.51 (8)	24.24 (8)	24.18 (8)	25.35 (9)	25.49 (9)	25.61 (9)	25.20 (9)	24.74 (4)
*M*(3) SS	12.03 (6)	12.10 (6)	12.19 (6)	12.19 (6)	12.17 (6)	11.96 (6)	11.95 (6)	12.56 (6)	12.59 (6)	12.66 (7)	12.40 (6)	12.06 (3)
Σ_*M*(1)+*M*(2)+*M*(3) SS_	60.5 (2)	60.9 (2)	61.4 (2)	61.3 (2)	61.2 (2)	60.3 (2)	60.3 (2)	63.2 (2)	63.5 (2)	63.9 (2)	62.6 (2)	61.05 (10)
*M*(4) SS	39.58 (9)	40.07 (10)	40.74 (10)	40.50 (10)	40.27 (10)	40.01 (9)	39.82 (9)	39.94 (10)	40.57 (10)	41.20 (10)	40.24 (9)	39.76 (4)
*A*(*m*) SS	5.03 (7)	5.40 (7)	5.78 (8)	5.58 (7)	5.54 (7)	5.59 (7)	5.54 (7)	4.08 (7)	4.38 (7)	4.75 (8)	4.50 (7)	3.82 (5)
〈*T*(1)—O〉	1.640	1.640	1.640	1.640	1.640	1.640	1.639	1.641	1.641	1.642	1.640	1.6369
〈*T*(2)—O〉	1.640	1.642	1.643	1.641	1.641	1.638	1.638	1.642	1.641	1.643	1.638	1.6337
〈*M*(1)—O〉	2.078	2.078	2.079	2.080	2.080	2.083	2.082	2.071	2.071	2.072	2.075	2.0729
〈*M*(2)—O〉	2.057	2.057	2.058	2.058	2.057	2.061	2.061	2.049	2.051	2.051	2.055	2.0603
〈*M*(3)—O〉	2.073	2.074	2.074	2.077	2.076	2.077	2.077	2.068	2.069	2.068	2.072	2.0636
〈〈*M*(1,2,3)—O〉〉	2.070	2.070	2.070	2.072	2.071	2.074	2.074	2.063	2.064	2.063	2.067	2.0655
〈〈*r* ^*M*(1,2,3)^〉〉†	0.711	0.712	0.712	0.714	0.713	0.716	0.716	0.703	0.704	0.704	0.708	0.706
〈*M*(4)—O〉	2.501	2.498	2.495	2.499	2.497	2.500	2.499	2.507	2.506	2.503	2.507	2.4961
〈Δσ_*M* SS_〉	2.75	2.70	5.51	4.21	3.44	3.64	3.82	7.74	9.63	12.34	6.40	
〈Δσ_BD_〉	3.31	3.34	3.41	3.51	3.46	3.57	3.43	3.86	3.80	4.01	3.83	

† Calculated as in Table 7 of Hawthorne & Oberti (2007 ▸).

**Table 7:** Average values of the spherical harmonic coefficients (Järvinen, 1993 ▸) used for modelling preferred orientation for all refinements

*y*20	0.014 (6)	*y*60	−0.022 (15)
*y*22*m*	0.015 (5)	*y*62*m*	0.004 (9)
*y*22*p*	0.072 (3)	*y*62*p*	0.019 (12)
*y*40	−0.009 (8)	*y*64*m*	0.021 (12)
*y*42*m*	0.011 (5)	*y*64*p*	0.001 (9)
*y*42*p*	0.0084 (19)	*y*66*m*	0.055 (6)
*y*44*m*	0.100 (2)	*y*66*p*	−0.042 (5)
*y*44*p*	0.041 (7)		
